# Effect of β-Cyclodextrin Complexation on Solubility and Enzymatic Conversion of Naringin

**DOI:** 10.3390/ijms131114251

**Published:** 2012-11-05

**Authors:** Li Cui, Zhen-Hai Zhang, E Sun, Xiao-Bin Jia

**Affiliations:** 1 Key Laboratory of New Drug Delivery System of Chinese Meteria Medica, Jiangsu Provincial Academy of Chinese Medicine, 100 Shizi Road, Nanjing 210028, Jiangsu, China; E-Mails: cuili2008516@126.com (L.C.); davidpharm@yeah.net (Z.-H.Z.); sune0825@yahoo.com.cn (ES.); 2 Nanjing University of Chinese Medicine, Nanjing 210046, Jiangsu, China

**Keywords:** β-cyclodextrin, inclusion complex, enzymolysis, naringin, naringenin

## Abstract

In the present paper, the effect of β-cyclodextrin (β-CD) inclusion complexation on the solubility and enzymatic hydrolysis of naringin was investigated. The inclusion complex of naringin/β-CD at the molar ratio of 1:1 was obtained by the dropping method and was characterized by differential scanning calorimetry. The solubility of naringin complexes in water at 37 ± 0.1 °C was 15 times greater than that of free naringin. Snailase-involved hydrolysis conditions were tested for the bioconversion of naringin into naringenin using the univariate experimental design. Naringin can be transformed into naringenin by snailase-involved hydrolysis. The optimum conditions for enzymatic hydrolysis were determined as follows: pH 5.0, temperature 37 °C, ratio of snailase/substrate 0.8, substrate concentration 20 mg·mL^−1^, and reaction time 12 h. Under the optimum conditions, the transforming rate of naringenin from naringin for inclusion complexes and free naringin was 98.7% and 56.2% respectively, suggesting that β-CD complexation can improve the aqueous solubility and consequently the enzymatic hydrolysis rate of naringin.

## 1. Introduction

**Figure 1:**
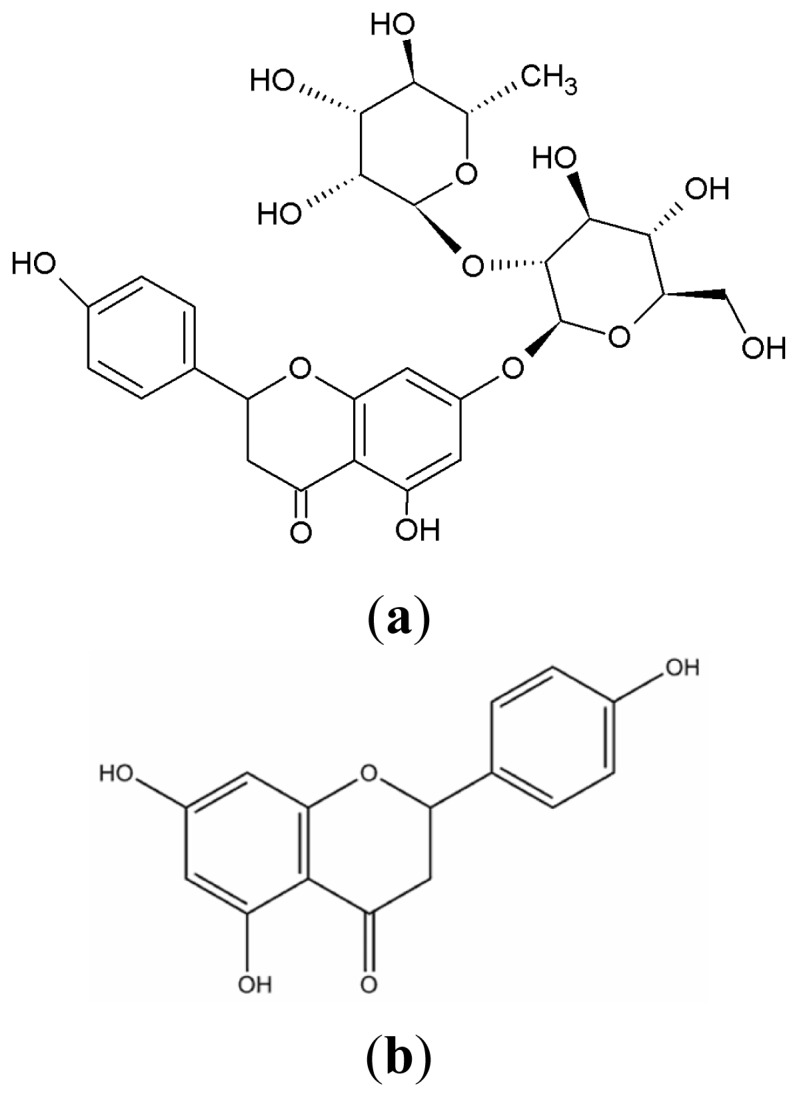
Chemical structures of naringin (**a**) and naringenin (**b**).

Naringenin (4′,5,7-trihydroxy-flavanone-7-rhamnoglucoside-glucopyranoside) is one of the phenolic flavonoids present in citrus fruits and grapefruit. Intervention with naringenin offers important biological/pharmacological antiatherogenic, hepatoprotective, nephroprotective, antiulcer, antioxidant, antiinflammatory, antimutagenic, vasodilator, antithrombotic, and anticancer activities. These restrain the hyperplasia of breast cancer and delay mammary tumorigenesis [1–4]. It has been reported that the content of natural naringenin is very low, which hinders further studies on its pharmacological actions [5]. Based on the close structural relationship (Figure 1) and the advantage of a relatively high content of natural naringin, naringenin might be obtained by enzymatic hydrolysis of naringin [6].

Numerous studies have been carried out to optimize the conditions to increase enzymatic hydrolysis rate. Helder J. Vila Real *et al.*[7] reported that the temperature of 30 °C was desirable for promoting the activity of the naringinase at 160 MPa, compared to atmospheric pressure. For the reaction rate, the pressure had a positive effect with a value of −15.0 ± 1.8 cm^3^·mol^−1^ for the activation volume. Helder and Pedro *et al.*[8] studied the effect of pressure in an enzymatic reaction with an immobilized biocatalyst. The result confirmed that pressure has a positive impact on naringin hydrolysis by immobilized naringinase in Ca-alginate beads with a negative activation volume of −9 mL·mol^−1^. Ribeiro *et al.*[6] suggested that there were many advantages in large scale processing by immobilizing biocatalysts. One of the simplest methods of immobilization is entrapment, which consists of the inclusion of enzymes or cells within polymeric matrices.

However, using the above measures, the enzymatic hydrolysis rate did not increase significantly. This may be owing to the fewer opportunities for naringin, a water-insoluble compound, to have sufficient contact with the enzyme. Moreover, high pressure was not cost-effective and the immobilized enzymes generally presented mass transfer limitations and showed lower catalytic activity due to reduced substrate transfer resistance [6]. Therefore, finding an approach that can increase the aqueous solubility of naringin and consequently increase the chances of contact with the enzyme might be a valuable strategy to increase the enzymatic hydrolysis rate of naringin.

Beta-cyclodextrin (β-CD) is a cyclic oligosaccharide containing seven glucopyranose units connected by α-(1,4) bonds [9]. On account of its structure and the relatively lipophilic surface of the internal cavity, which is in contrast with the hydrophilic feature of the external hydroxyl faces, β-CD molecules can easily form inclusion complexes with a wide variety of molecules and molecular ions [10–13]. In the pharmaceutical industry, β-CD has often been used to enhance the solubility of drugs, such as indomethacin, naringin, celecoxib, and citric acid [14–17]. This lets us speculate that preparing naringin inclusion complexes may increase the solubility and the enzymatic hydrolysis rate of naringin.

Snailase is an enzyme extracted from crops or enteron of snails, including complex cellulases (endoglucanase, exoglucanase, and β-glucosidase) and β-galactosidase. This enzyme has long been recognized as potentially useful due to its high activity and low operating temperatures [18,19].

To the best of our knowledge, no attempt has been made to prepare naringenin from naringin inclusion complex using snailase. The main objectives of the present study were to evaluate the effect of β-CD on the aqueous solubility and hydrolysis rate of naringin, and to identify the key parameters (pH value, temperature, ratio of substrate/enzyme, concentration of the substrate, and reaction time) for the enzymatic hydrolysis with a univariate experimental design.

## 2. Results and Discussion

### 2.1. Differential Scanning Calorimetry

**Figure 2:**
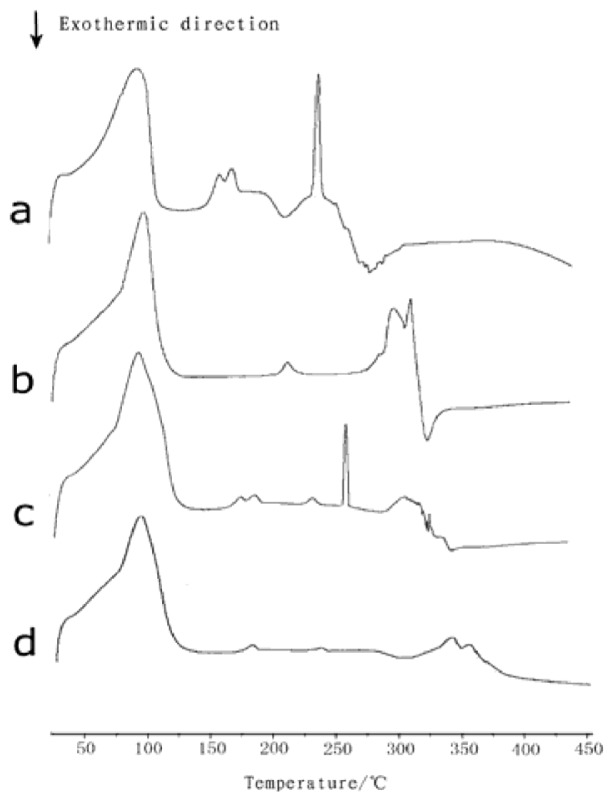
Differential scanning calorimetric (DSC) curves: (**a**) naringin; (**b**) β-cyclodextrin (β-CD); (**c**) equimolecular physical mixture of naringin and β-CD; (**d**) inclusion complex of naringin.

Differential scanning calorimetric (DSC) analysis was generally performed to characterize inclusion complexes with β-CD by comparing the thermal behaviors of the individual components as well as their physical mixtures and inclusion compounds [20–22]. DSC is a rapid and reliable way to screen naringin and β-CD compatibility. The DSC curves of naringin, β-CD, naringin/β-CD physical mixture, and inclusion complex are shown in Figure 2a–d. Naringin is a crystalline compound, showing an exothermic peak at its melting temperature (approximately 250 °C), followed by its decomposition at 300 °C. The dehydration and decomposition of β-CD occurred at approximately 100 °C and 320 °C, respectively. The physical mixture of naringin and β-CD obtained the same exothermic peak as that of naringin. However, this characteristic peak of naringin melt was not observed for the naringin–β-CD complex, indicating that the interaction of naringin molecules to form a crystal structure was destroyed by β-CD in the naringin–β-CD complex. These DSC results confirmed that naringin was no longer present as a crystalline material and its β-CD solid complexes existed in the amorphous state because a β-CD molecule encapsulated each naringin molecule^21^. The result was in accordance with the data in previous studies [23].

### 2.2. Analytical Method Validation

High performance liquid chromatography (HPLC) analysis showed that good linear relationships between concentrations and the peak areas of naringin and naringenin were obtained within the range of 1.96 to 31.36 μg·mL^−1^ and 0.30 to 9.62 mg·mL^−1^, respectively. The regression equations were as follows:

(1) $$\text{naringin}:Y_{1}=20957X_{1}+732.89\;\;\;r_{1}=0.9995$$

(2) $$\text{naringenin}:Y_{2}=1.42\times 10^{6}\;X_{2}-1236.2\;\;\;r_{2}=0.9998$$

### 2.3. Solubility of Inclusion Complex

The data were calculated by Equation (1), it was found that at 37 ± 0.1 °C, the solubility of naringin in the inclusion complex was 28.5 μg·mL^−1^, whereas the value of free naringin was only 1.9 μg·mL^−1^. The solubility of the naringin complex in water was nearly 15 times greater than that of free naringin, indicating that β-CD complexation significantly increased the solubility of naringin.

### 2.4. Univariate Experiments

The inclusion complex and free naringin can be transformed into naringenin by snailase. For the inclusion complex and free naringin, the main parameters that affected enzymatic hydrolysis were pH value, temperature, ratio of snailase/substrate, concentration of the substrate and reaction time.

#### 2.4.1. Effect of pH Value on Enzymatic Hydrolysis of the Inclusion Complex and Free Naringin

**Figure 3:**
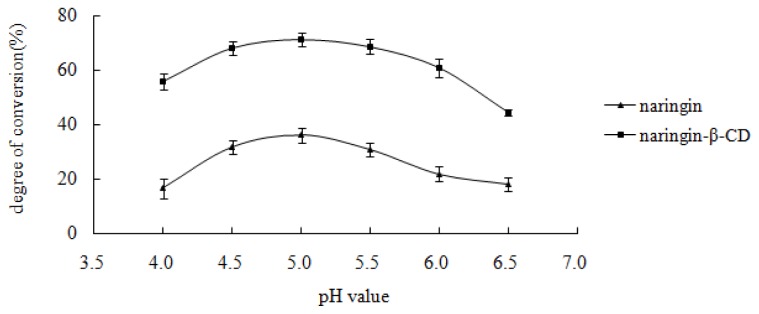
Effect of pH value of acetate buffer. Reaction was performed by adding 10 mg substrate, 10 mg snailase, and 1 mL glacial acetic acid and sodium acetate anhydrous buffer (pH 4.0, 4.5, 5.0, 5.5, 6.0, and 6.5) at 37 °C for 24 h. Each point represents the mean ± SD (*n* = 3).

As shown in Figure 3, the highest hydrolysis rate of the inclusion complex and free naringin was obtained at the buffer pH value of 5.0, thus the optimal pH value for the hydrolysis of inclusion complex and free naringin was five.

#### 2.4.2. Effect of Temperature on Enzymatic Hydrolysis of the Inclusion Complex and Free Naringin

**Figure 4:**
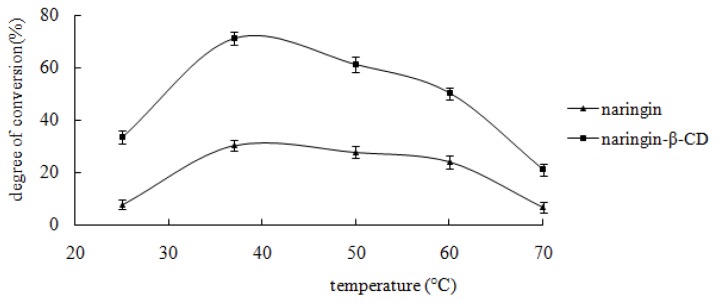
Effect of reaction temperature. Ten milligrams of substrate, 10 mg snailase, and 10 mL glacial acetic acid and anhydrous sodium acetate buffer (pH 4.5) were mixed in a 1 mL Eppendorf tube. Subsequently, the buffer was incubated in a HH-4 digital constant temperature water bath at 25, 37, 50, and 60 °C for 24 h. Each point represented the mean ± SD (*n* = 3).

The influence of temperature on the bioconversion in the present study was performed within a range of 25–70 °C. Results are shown in Figure 4. From Figure 4, it was found that the highest hydrolysis rate of the inclusion complex and free naringin was obtained at 37 °C. Thus, the optimal temperature for hydrolysis of both the inclusion complex and free naringin was 37 °C.

Numerous studies described temperature as a parameter with an optimal value in enzymatic processes [24,25]. The optimal temperature for the inclusion complex and free naringin was the same, indicating that β-CD had no influence on the enzymatic temperature.

#### 2.4.3. Effect of Snailase/Substrate Ratio on Enzymatic Hydrolysis of the Inclusion Complex and Free Naringin

**Figure 5:**
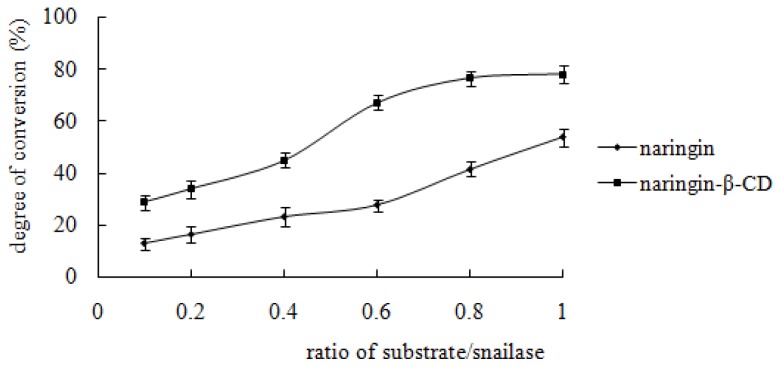
Effect of the ratio of snailase/substrate. Nineteen point six milligrams of inclusion complex (containing 10 mg naringin) or 10 mg free naringin was mixed with 1, 2, 4, 6, 8, 10 mg snailase (snailase/substrate = 0.1, 0.2, 0.4, 0.6, 0.8, and 1). Glacial acetic acid and sodium acetate anhydrous buffer (pH 4.5) was added to the mixture at a final volume of 1 mL. Subsequently, the buffer was incubated in a HH-4 digital constant temperature water bath at 37 °C for 24 h. Each point represented the mean ± SD (*n* = 3).

From Figure 5, it can be seen that the hydrolysis percentage of the inclusion complex is almost constant when the ratio of snailase/substrate is between 0.8 and 1, whereas the hydrolysis percentage of free naringin increases linearly with the increase of the ratio of snailase/substrate. Based on these data, 0.8 and 1 were identified as the optimal ratio of snailase/substrate for hydrolysis of the inclusion complex and free naringin, respectively.

#### 2.4.4. Effect of the Substrate Concentration on Enzymatic Hydrolysis of the Inclusion Complex and Free Naringin

**Figure 6:**
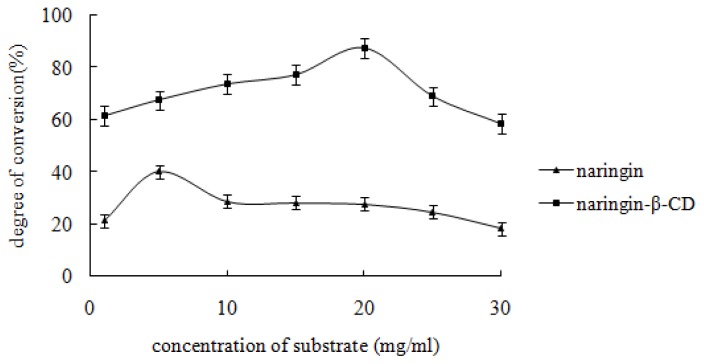
Effect of the concentration of the substrate. The substrate and snailase were weighed according to the weight proportion of 1:1. Glacial acetic acid and sodium acetate anhydrous buffer (pH 4.5) were added and the following solution concentrations were obtained: 1, 5, 10, 20, and 30 mg·mL^−1^. Subsequently, the buffer was incubated in a HH-4 digital constant temperature water bath at 37 °C for 24 h. Each point represents the mean ± SD (*n* = 3).

The influence of the substrate concentration was evaluated at pH 5.0 and 37 °C with a reaction time of 24 h. The results are demonstrated in Figure 6. The hydrolysis percentage of the inclusion complex or free naringin increased with the concentration rising, and subsequently dropped slightly. The highest hydrolysis percentage for inclusion complex or free naringin was achieved at a substrate concentration of 20 mg·mL^−1^ and 5 mg·mL^−1^ respectively.

#### 2.4.5. Effect of the Reaction Time on Enzymatic Hydrolysis of the Inclusion Complex and Free Naringin

**Figure 7:**
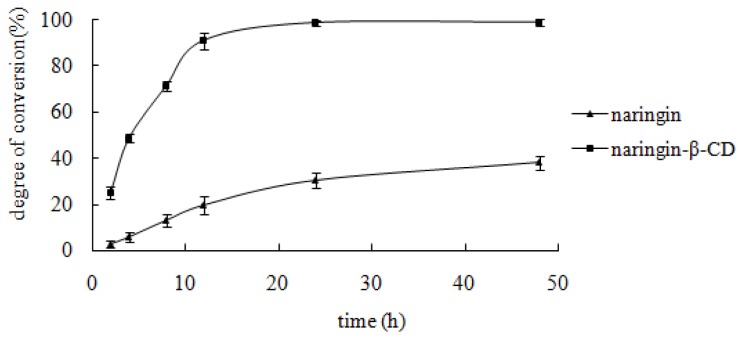
Effect of reaction time. Ten milligrams of substrate, 10 mg snailase, and 1 mL glacial acetic acid and sodium acetate anhydrous buffer (pH 4.5) were mixed in a 1 mL Eppendorf tube. Subsequently, the buffer was incubated in a HH-4 digital constant temperature water bath at 37 °C for 2, 4, 8, 12, 24, and 48 h. Each point represents the mean ± SD (*n* = 3).

Figure 7 shows the effect of reaction time on enzymatic hydrolysis. The hydrolysis percentage of both inclusion complex and free naringin increased with the ongoing reaction time. A value, which was near the maximum, was obtained at 12 h for the inclusion complex and at 48 h for naringin.

The reaction time was an important factor for snailase-involved hydrolysis [26]. From 0 to 12 h, the hydrolysis speed of the inclusion complex was considerably faster than that of free naringin (*p* < 0.001). A gradual increase in speed was observed. At the 12 h point, the hydrolysis percentage of the inclusion complex was greater than 90%, whereas percentage of free naringin was approximately 20%.

From all the above univariate experiments, the optimal conditions for snailase involved hydrolysis were found to be as follows: pH 5.0, temperature 37 °C, ratio of snailase/substrate 0.8, substrate concentration 20 mg·mL^−1^, and reaction time 12 h. Under the optimal reaction conditions, the hydrolysis percentage of the naringin inclusion complex and free naringin were 98.7% and 56.2%, respectively, suggesting that β-CD inclusion complexation could significantly increase the enzymatic hydrolysis rate of naringin.

## 3. Experimental Section

### 3.1. Materials and Equipment

Standard naringin (98% HPLC) and naringenin (98% HPLC) were purchased from Xi’an Xiao Cao Botanical Development Co., Ltd. (Xi’an, China), the snailase from Baier Di Biotechnology Co., Ltd. (Beijing, China), β-cyclodextrin (average *M*_w_ = 1135) from Shanghai Chemical Reagent Company of China Pharmaceutical Group, and glacial acetic acid, anhydrous sodium acetate and absolute ethanol from Nanjing Chemical Reagent Co., Ltd. Acetonitrile was of HPLC grade. All the other reagents were of analytical grade. The ultra-pure water was purified by the Milli-Q water purification system (Millipore, Bedford, MA, USA).

All quantitative analyses were carried out by a high-performance liquid chromatographic system composed of the Waters 2690 Separation Module (comprising in-line degasser, quaternary solvent delivery pumps, automatic injector, and a column oven) with a Photodiode Array Detector (Model Waters 2996) and Phenomenex^®^ C18 column (250 × 4.60 mm, Phenomen Tech Co, Ltd.). The enzymatic hydrolysis was carried out by a digital constant temperature water bath HH-4 (Guohua Electric Appliance Co., Ltd. Shenzhen, China). A TGL-16H high-speed centrifuge (Shanghai Precision Instrument Factory Anping, Shanghai, China) was employed to treat the samples. DSC204F1 differential scanning calorimeter (Germany) was used to confirm the complexation of naringin. Bruker Avance 300 NMR spectrometer (Swiss Avance) was used for structure identification.

### 3.2. Preparation and Characterization of Inclusion Complex of Naringin

#### 3.2.1. Preparation of Inclusion Complex

The inclusion complex of naringin with β-CD at 1:1 molar ratio was prepared using the dropping method. The accurately weighed β-CD was dissolved in distilled water to get a saturated solution. Then the naringin solution in absolute ethanol was added drop by drop and a suspension was formed. The suspension was agitated for 2 h at 30 °C. Subsequently, it was placed in a cold storage for 24 h. The suspension was filtered and free naringin was washed from the residue using ethanol. Finally, the residue was dried at 100 °C for 1 h on a water bath.

#### 3.2.2. Preparation of the Physical Mixture of Naringin and β-CD

A physical mixture of naringin and β-CD in the same weight ratio as the co-evaporated complex was prepared. The individual components of the physical mixture were first sieved through a 315 μm mesh in a mortar and pestle for 5 min.

#### 3.2.3. Differential Scanning Calorimetry (DSC)

To obtain the DSC curves of naringin, β-CD of the equimolecular physical mixtures of naringin and inclusion complex, a Photo DSC204F1 differential scanning calorimeter calibrated with indium was utilized. Accuratel weighed samples (1–5 mg) were placed in a 40 μL aluminum pan, which was sealed and pierced. Aluminum was used as a reference material and the scanning rate was 10.00 °C·min^−1^ with a scanning temperature range of 0–450 °C. Measurements were made in duplicate for each sample.

### 3.3. Analytical Method Validation

#### 3.3.1. The Calibration Curve of Naringin

About 5mg of naringin (purity 98%) was accurately weighted and dissolved with 50 mL absolute ethanol to get store solutions. Then 0.5, 1.0, 1.5, 2.0, 2.5, and 3.0 mL stock solutions were taken and diluted with ethanol to achieve a final volume of 25 mL. The working solutions were analyzed by the HPLC system with an isocratic elution of acetonitrile-H_2_O containing 0.1% formic acid (38:62, *v*/*v*) as mobile phase at a flow rate of 1.0 mL·min^−1^, and detected at 285 nm. The regression equation was derived from the values between the peak area (*Y*_1_) and the concentrations (*X*_1_) of naringin. The content of naringin was calculated with the regression equations.

#### 3.3.2. The Calibration Curve of Naringenin

About 20 mg of naringenin (purity 98%) was accurately weighed and dissolved with 2 mL absolute ethanol to get the stock solution. Then 0.03, 0.06, 0.12, 0.24, 0.48, and 0.96 mL stock solution was taken and diluted with absolute ethanol to achieve a final volume of 1 mL. The working solutions were analyzed as described in Section 2.3.1. The regression equation was derived from the values between the peak area (*Y*_2_) and the concentrations (*X*_2_) of naringenin. The content of naringenin was calculated with the regression equations.

### 3.4. Calculation of Conversion

After reaction, 100 μL sample solution was taken and diluted with 900 μL absolute ethanol, vortexed for 60 s, then centrifuged at 11,000 rpm for 10 min, 20 μL supernatant was injected into HPLC. The degree of conversion was calculated by the following equation:

(3) $$\text{Degree of conversion }(\%)=[CV/(m\times M_{\text{W}1}/M_{\text{W}2})]\times 100\%$$

*C*—concentration of naringenin, *V*—volume of naringenin, *m*—weight of naringin, *M*_W1_—molecular weight of naringin, *M*_W2_—molecular weight of naringenin.

### 3.5. Solubility of Inclusion Complex

Solubility measurements were carried out as described below. Excess amounts of the inclusion complex or free naringin were added to 10 mL of aqueous solutions. The suspensions were shaken for 72 h at 37 ± 0.1 °C with a constant speed of 150 r·min^−1^. The suspensions were protected from light to achieve equilibration. After equilibration, the suspensions were filtered through 0.45 μm membranes. The first 15% of the filtrate was abandoned to avoid any potential loss of the drug because of absorption by the filter. The subsequent filtrate was collected. All procedures were conducted at 37 ± 0.1 °C to avoid any precipitation of the drug. The filtrate was appropriately diluted by ethanol and the concentration of the substrate in the filtrate was determined by the HPLC system.

### 3.6. Univariate Experiment for Naringin-β-CD and Naringin

Nineteen point six milligrams of inclusion complex (containing 10 mg naringin) or 10 mg free naringin and 10 mg snailase were suspended in a 1 mL buffer system comprising glacial acetic acid and anhydrous sodium acetate. The conditions of enzymatic hydrolysis for the inclusion complex of naringin and free naringin into naringenin were tested, including pH value, temperature, ratio of enzyme/substrate, concentration of the substrate, and reaction time. To evaluate the effect of each factor on enzymatic hydrolysis, the other four factors were fixed, and all the reactions were tested in triplicate. The final reaction product was dried with the N_2_ blow and dissolved in ethanol. Subsequently, 10 μL filtrated solution was injected into the HPLC system. The conversion rate of naringin in each reaction was used to reflect the enzymatic hydrolysis efficiency.

## 4. Conclusions

β-CD inclusion complexation can significantly increase the solubility of naringin in water and consequently increase the enzymatic hydrolysis rate of naringin to generate naringenin.

## References

[1] Ali G., Hawa Z.E.J., Asmah R. (2010). Synthesis of Phenolics and Flavonoids in Ginger (*Zingiber officinale* Roscoe) and Their Effects on Photosynthesis Rate. Int. J. Mol. Sci.

[2] Raja N.Z.R.A.R., Iffah I.Z., Abu B.S., Mahiran B. (2012). Enzymatic Properties and Mutational Studies of Chalcone Synthase from Physcomitrella patens. Int. J. Mol. Sci..

[3] Chen H.J., Baskaran S.I., Chen B.H. (2012). Determination of Phenolic Acids and Flavonoids in Taraxacum formosanum Kitam by Liquid Chromatography-Tandem Mass Spectrometry Coupled with a Post-Column Derivatization Technique. Int. J. Mol. Sci.

[4] Badary O.A., Maksoud S.A., Ahmed W.A., Owieda G.H. (2005). Naringenin attenuates cisplatin nephrotoxicity in rats. Life Sci.

[5] Erlund I. (2004). Review of the flavonoids quercetin, hesperetin, and naringenin. Dietary sources, bioactivities, bioavailability, and epidemiology. Nutr. Res.

[6] Isabel A.C., Maria H.L.R. (2008). Kinetic modelling of naringin hydrolysis using a bitter sweet alfa-rhamnopyranosidase immobilized in k-carrageenan. J. Mol. Catal. B.

[7] Vila Real H.J., Alfaia A.J., Calado A.R.T., Ribeiro M.H.L. (2007). High pressure-temperature effects on enzymatic activity:Naringin bioconversion. Food Chem.

[8] Pedro H.A.L., Alfaia A.J., Marques J., Vila-Real H.J., Calado A., Ribeiro M.H.L. (2007). Design of an immobilized enzyme system for naringin hydrolysis at high-pressure. Enzyme Microb. Technol.

[9] Loftsson T., Brewster M.E. (1996). Pharmaceutical applications of cyclodextrins. Drug solubilization and stabilization. J. Pharm. Sci.

[10] Anca H., Cristina P., Aurel A., Miruna S., Nicoleta H., Mihali C.V., Marieta C., Anca D. (2012). Hepatoprotective Effects of *Berberis vulgaris* L. Extract/β Cyclodextrin on Carbon Tetrachloride–Induced Acute Toxicity in Mice. Int. J. Mol. Sci.

[11] Sharifah M., Hemavathy S., Muggundha R., Tilagam M., Kumuthini C., Puvaneswary S. (2011). Conventional Study on Novel Dicationic Ionic Liquid Inclusion with β-Cyclodextrin. Int. J. Mol. Sci.

[12] Sun T., Jiang B., Pan B.L. (2011). Microwave Accelerated Transglycosylation of Rutin by Cyclodextrin Glucanotransferase from *Bacillus* sp. SK13.002. Int. J. Mol. Sci.

[13] Ratnasooriya C.C., Rupasinghe H.P.V. (2012). Extraction of phenolic compounds from grapes and their pomace using beta-cyclodextrin. Food Chem.

[14] Casella R., Williams D.A., Jambhekar S.S. (1998). Solid-state β-cyclodextrin complexes containing indomethacin, ammonia and water. II. Solubility studies. Int. J. Pharm.

[15] Divakar S. (1993). A structural study of the naringin-β-cyclodextrin complex. J. Incl. Phenom. Macrocycl. Chem.

[16] Swati R., Sanjay K.J. (2004). Solubility enhancement of celecoxib using β-cyclodextrin inclusion complexes. Eur. J. Pharm. Biopharm.

[17] Germain P., Bilal M., de Brauer C. (1995). Beta-cyclodextrin/citric acid complexation equilibrium thermodynamic study. Apparent solubility of β-CD in aqueous solutions of citric acid. Thermochim. Acta.

[18] Flari V., Matoub M., Rouland C. (1995). Purification and characterization of a b-mannanase from the digestive tract of the edible snail *Helix lucorum* L. Carbohydr. Res..

[19] Got R., Marnay A., Jarrige P., Font J. (1964). Beta-galactosidase of *Helix pomatia*. Nature.

[20] Bilensoy E., Cırpanlı Y., Sen M., Doğan A.L., Calıs S. (2007). Thermosensitive mucoadhesive gel formulation loaded with 5-Fu: Cyclodextrin complex for HPV-induced cervical cancer. J. Incl. Phenom. Macrocycl. Chem.

[21] Calderini A., Pessine F.B.T. (2008). Synthesis and characterization of inclusion complex of the vasodilator drug minoxidil with beta-cyclodextrin. J. Incl. Phenom. Macrocycl. Chem.

[22] Cevher E., Sensoy D., Zloh M., Mulazimoglu L. (2008). Preparation and characterisation of natamycin: γ-Cyclodextrin inclusion complex and its evaluation in vaginal mucoadhesive formulations. J. Pharm. Sci.

[23] Sansone F., Picerno P., Mencherini T. (2011). Flavonoid microparticles by spray-drying: Influence of enhancers of the dissolution rate on properties and stability. J. Food Eng.

[24] Liaset B., Julshamn K., Espe M. (2003). Chemical composition and theoretical nutritional evaluation of the produced fractions from enzymic hydrolysis of salmon frames with Protamex™. Process Biochem.

[25] Rosenthal A., Pyle D., Niranjan K., Gilmour S., Trinca L. (2001). Combined effect of operational variables and enzyme activity on aqueous enzymatic extraction of oil and protein from soybean. Enzyme Microb. Technol.

[26] You J.Y., Peng C., Liu X. (2011). Enzymatic hydrolysis and extraction of arachidonic acid rich lipids from *Mortierella alpine*. Bioresour. Technol.

